# A single dose of purple grape juice improves physical performance and antioxidant activity in runners: a randomized, crossover, double-blind, placebo study

**DOI:** 10.1007/s00394-019-02139-6

**Published:** 2019-11-15

**Authors:** Lydiane de Lima Tavares Toscano, Alexandre Sérgio Silva, Ana Carla Lima de França, Bruno Rafael Virgínio de Sousa, Eder Jackson Bezerra de Almeida Filho, Matheus da Silveira Costa, Aline Telles Biasoto Marques, Darcilene Fiuza da Silva, Klécia de Farias Sena, Gilberto Santos Cerqueira, Maria da Conceição Rodrigues Gonçalves

**Affiliations:** 1 Programa de Pós-graduação em Ciências da Nutrição, Universidade Federal da Paraíba (UFPB), João Pessoa, Paraíba Brazil; 2 Laboratório de Estudos do Treinamento Físico Aplicado ao Desempenho e a Saúde, Departamento de Educação Física, Universidade Federal da Paraíba (UFPB), Centro de Ciências da Saúde, Campus I, Cidade Universitária, João Pessoa, Paraíba CEP 58059-900 Brazil; 3 Empresa Brasileira de Pesquisa Agropecuária, Embrapa Semiárido, Petrolina, Pernambuco Brazil; 4 Faculdade de Farmácia, Universidade Federal da Bahia, Salvador, Bahia Brazil; 5 Departamento de Morfologia, Universidade Federal do Ceará, Fortaleza, Ceará Brazil

**Keywords:** Polyphenols, Ergogenic substances, Sports nutrition, Athletic performance, Oxidative stress

## Abstract

**Purpose:**

To investigate the effects of a single dose of juice on physical performance, oxidative stress, inflammation and muscle damage in runners.

**Methods:**

Fourteen recreational male runners (39 ± 9 years, *V*O_2peak_ = 55.9 ± 6.5 ml/kg/min) performed two running tests to exhaustion at 80% of *V*O_2max_ after ingesting grape juice or a placebo drink (10 ml/kg/day) randomly. Blood samples were taken before and 2 h after supplementation and immediately after running to analyze total antioxidant capacity (TAC), malondialdehyde (MDA), alpha-1 acid glycoprotein (A1GPA), high-sensitivity C-reactive protein (hs-CRP), creatine kinase (CK) and lactate dehydrogenase (LDH).

**Results:**

The participants ran for an average of 59.2 ± 27.8 min until exhaustion in the placebo group and for 68.4 ± 29.7 min until exhaustion in the grape juice intake group, which was a significantly longer time (*p* = 0.008). This improvement in physical performance was accompanied by a 43.6% increase in TAC (*p* = 0.000) at the post-exercise timepoint compared to the level at baseline. MDA, A1GPA, hs-CRP, CK, and LDH did not exhibit changes. In contrast, no significant change in any variable was observed after consuming the placebo drink.

**Conclusion:**

The single-dose intake of purple grape juice demonstrated an ergogenic effect in recreational runners by increasing run time to exhaustion and increasing antioxidant activity.

## Introduction

Purple grapes and their derivatives have been recognized as foods that have beneficial effects on blood pressure [1], vascular density [2], neurocognitive function [3] and lipoprotein metabolism [4]. In addition, purple grapes are able to protect against oxidative stress induced by both diseases [5] and strenuous exercise [6]. This protective effect has been attributed to purple grape juice because it is rich in antioxidants such as flavonoids (flavanois, flavonois and anthocyanins) and nonflavonoids (phenolic acids and resveratrol) [7].

Cardiometabolic benefits of purple grapes have been expanded to the athletic context. In animal models, grape products promoted improvements in physical performance [8–10], antioxidant protection [11, 12] and anti-inflammation [10]. In a previous study in our laboratory, purple grape juice ingestion (10 mL/kg/day for 28 days) promoted a 15% increase in time to exhaustion in a running test, which was accompanied by increased antioxidant activity and reduced inflammation in recreational runners [13]. In the athletic context, this improvement in physical performance is relevant.

While in the previous study the ergogenic effect was observed after almost a month of nutritional intervention, there is evidence indicating that single doses of various foods, such as polyphenols [14–17], beetroot juice [18] and chocolate milk [19], promoted improved athletic performance. In addition, single dose supplementation with a polyphenol-rich extract (green tea, grape and pomegranate) [17], dark chocolate [20] and antioxidant ice cream (dark cocoa powder, hazelnut and green tea extracts) [21] was able to improve oxidative stress biomarkers.

Based on this evidence, we investigated the acute ergogenic effect of purple grape juice on recreational runners, testing the hypothesis that ingestion of a single dose of grape juice may be sufficient to promote improvement in physical performance, an effect that was previously demonstrated when grape juice was chronically administered [13].

The objective of this study was to investigate the effects of the intake of a single dose of purple grape juice on physical performance, oxidative stress, inflammation and muscular fatigue in male recreational runners submitted to a running session until exhaustion.

## Methods

### Study type and participants

**Fig. 1:**
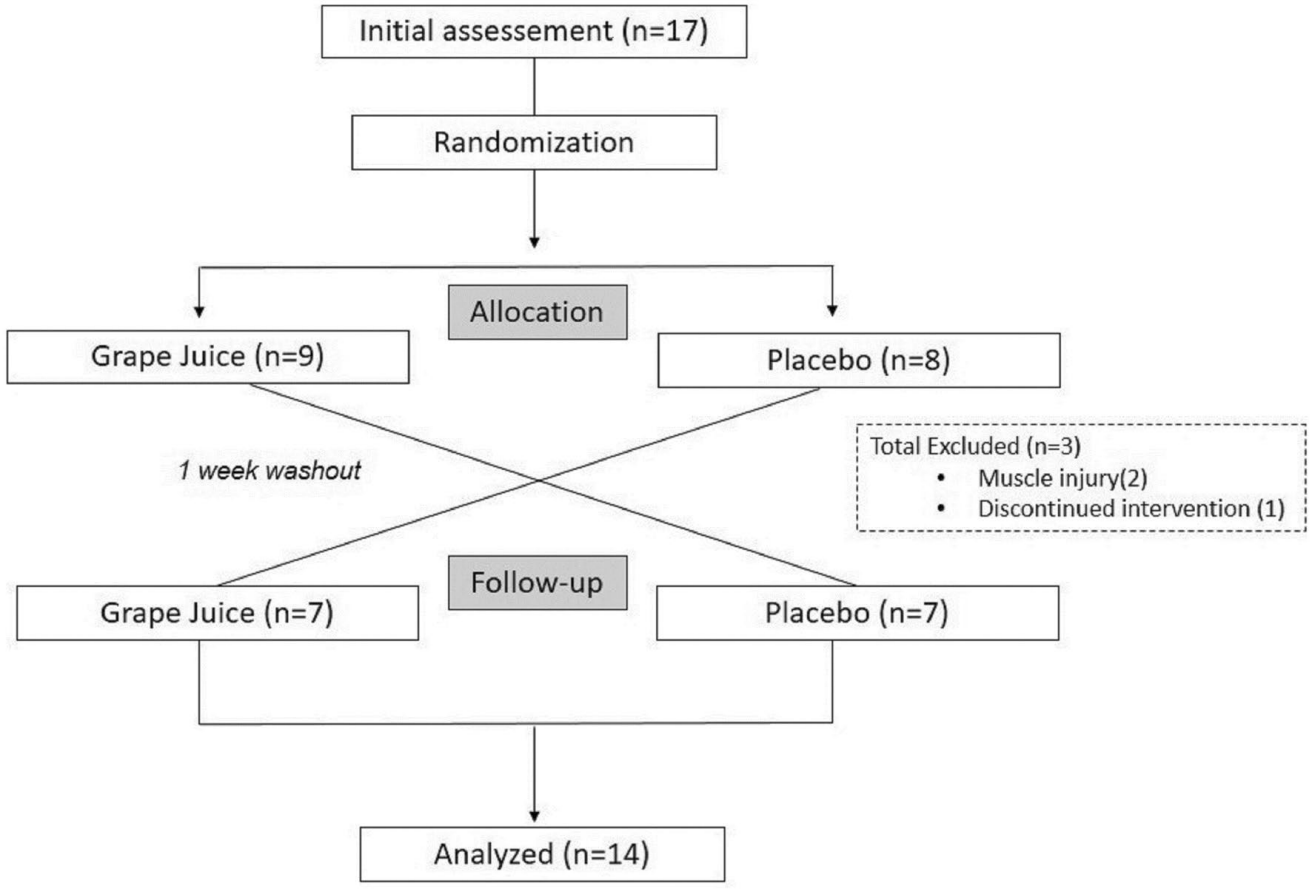
CONSORT flow diagram

A double-blind, crossover, randomized, placebo-controlled clinical trial was conducted with 14 male recreational runners. The sample size was established a posteriori; the effect size was calculated as *d* = 0.30 by the Cohen test, which revealed 12 participants as the minimum sample size considering an *α* error of 0.05 and a *β* error of 0.80. All the athletes performed the two procedures, one experimental and another control, in a randomized crossover model (www.randomizer.org). This information is presented in the flowchart (Fig. 1).

To participate in the study, athletes needed to have completed at least 1 year of training with a frequency of five training sessions per week (at least three sessions needed to be running) for at least 2 months without interruption, and the participants needed to be participating in competitions on a regular basis. The participants did not have any chronic degenerative diseases, did not smoke, and did not chronically use any medication. In addition, the participants did not have the habit of consuming red wine, purple grape juice, dietary supplements, vitamins, or bioactive grape products (polyphenols) regularly. Athletes who suffered from skeletal muscle injuries during the study, those who changed their usual eating or physical training patterns, those who started drug therapy, and those who did not consume the proper amounts of products provided during the study period were excluded.

### Experimental design

**Fig. 2:**
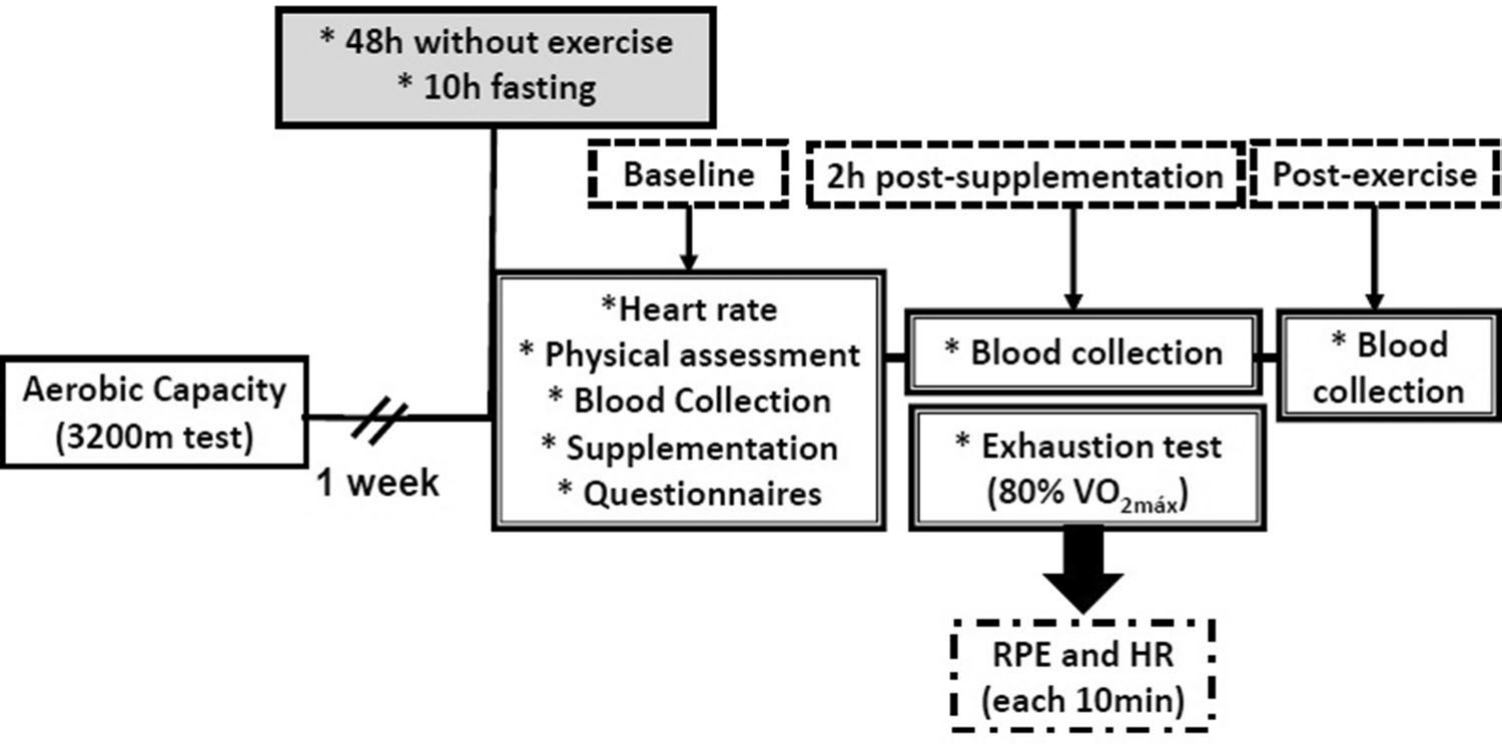
Design of the experimental study

Initially, athletes performed a 3200-m run test [22] to estimate aerobic capacity. In the following 2 weeks, they participated in the experimental procedures by performing the test run to exhaustion after supplementation (10 ml/kg/day of whole grape juice) or the placebo drink, and the two tests were separated by a week. As demonstrated in Fig. 2, after 48 h without training and overnight fast (10 h) [23], the athletes were submitted to nutritional, sleep and recovery assessment. Blood samples were collected before the ingestion of the grape juice or placebo drink, 2 h after ingestion, and immediately after the test run to exhaustion for further analysis of biomarkers of oxidative stress, inflammation and muscle injury.

### Preparation of participants for procedures

Athletes were instructed to abstain from exercise for the 48 h before the experimental sessions and performing overnight fast (10 h). Were instructed not to ingest any nutritional supplement or antioxidant foods rich in grape polyphenols during the study. In addition, no alcoholic beverages were ingested in the preceding 24 h, and caffeine was not consumed the night before the experiment to ensure the exclusion of any effects associated with diet. At six o’clock in the morning, after arriving at the laboratory, they were accommodated in a climatized room and seated at rest for 10 min to measure heart rate, and then baseline blood collection was performed. Subsequently, the athletes consumed a standardized snack consisting of a sandwich (50 g of wheat bread + 34 g of white processed cheese = 152 kcal, 21.8 g of carbohydrates, 7.4 g of protein, 3.6 g fat, 4.0 g fiber) with the experimental or placebo beverage, according to previous randomization.

### Nutritional assessment

Body fat percent was assessed in accordance with the protocol described by Jackson and Pollock [24] for men using a clinical plicometer (Sanny, São Paulo, Brazil). Dietary intake throughout the study was evaluated by 24-h dietary recall three times for each participant; two recalls corresponded to weekdays and the other represented a weekend day. Two of the dietary recalls were performed in the days prior to the experimental sessions. The average of the three dietary recalls was used to determine the nutritional profile. Other dietary recalls were applied to characterize the consumption in the 24 h preceding the experimental tests (Avanutri^®^, Rio de Janeiro, Brazil). In addition, possible gastrointestinal discomforts such as abdominal pain, bloating, constipation, diarrhea, heartburn, flatulence and nausea were reported.

### Characterization of grape juice and the placebo drink

The experimental product used for the supplementation was purple grape juice of the Cooperativa Vinícola Garibaldi (Garibaldi, Serra Gaúcha, Brazil) produced from grapes of the Isabel, Bordeaux, and Concord (*Vitis labrusca*) varieties. This product was characterized as a natural, integral (100% fruit), non-alcoholic beverage with no added sugar, water, flavoring or preservatives, according to the manufacturer’s information. The placebo drink (Aliança Premier) was a grape refreshment developed by Vinícola Nova Aliança (Flores da Cunha, Rio Grande do Sul, Brazil) for specific research purposes. According to the producer, 200 mL of the grape juice contains 130 kcal and 32 g of carbohydrates, while the placebo drink has 140 kcal and 33 g of carbohydrates. They do not contain significant amounts of proteins, total fats, saturated fats, trans fats, dietary fiber or sodium. Beverages were isovolumetric with similar caloric values and amounts of carbohydrates.

Samples of grape juice and the placebo beverage were sent to the Embrapa Semiarid Laboratory of Enology (Petrolina, Pernambuco, Brazil) and evaluated in triplicate. The antioxidant composition was determined by free radical scavenging activities using DPPH (2,2-diphenyl-1-picryl-hydrazyl) [25] and ABTS (2,2′-azinobis-3-ethylbenzthiazoline-6 sulfonic acid) [26, 27], while the total phenolic content was determined according to the methodology described by Folin-Ciocateau [28]. Quantification of the phenolic compounds (flavanois, flavonois, phenolic acids and stilbenes) was performed by high-performance liquid chromatography (HPLC) using a Waters 2695 Alliance system (Milford, MA, USA) equipped with a diode array detector (DAD) and a fluorescence detector (FLD) according to a method validated by EMBRAPA.

### Supplementation protocol

The athletes received 10 ml/kg/day [13] of the supplement 2 h before the run test until exhaustion so that they could increase the bioavailability of the polyphenolic compounds present in the experimental drink [29]. The volunteers and researchers involved in the procedures were blinded to supplementation. The experimental juice and placebo beverage had similar colors and flavors, as well as similar caloric values and carbohydrate contents.

### Aerobic capacity test (3200 m)

Maximum aerobic capacity was determined by means of the 3200-m run test. The test was performed individually for each athlete; they ran eight laps around a 400-m official athletics track. The athletes were instructed to start the test when they felt ready and to complete the course (3200 m) in the shortest possible time. The total execution time was measured with a stopwatch and recorded by the responsible researcher. The *V*O_2max_ was estimated using the following equation: *V*O_2max_ (ml/kg^−1^/min^−1^) = 118.4 − 4.774 × (*T*), where *T* = time in minutes with a decimal fraction to complete the 3200-m run [22].

### Subjective stress assessment and recovery/rest

In a quiet, climatized environment without external interference, the athletes were invited and oriented to complete three questionnaires to better characterize their previous conditions of stress and rest. These were applied twice for each athlete, always before each exhaustion test. The Profile of Mood States (POMS) [30] was used to evaluate the psychometric state from Total Humoral Disturbance (THD), the EPWORTH Sleepiness Scale-Brazilian (ESS-BR) [31] was used to evaluate the situations related to the occurrence of daytime sleepiness, and the Stress and Recovery Questionnaire for Athletes (RESTQ-Sport) [32] was used to evaluate the state of stress and recovery from everyday situations that are potentially stressful and restful.

### Run to exhaustion test

On the day of experiments, after 2 h of the standardized breakfast with the experimental or placebo beverage, the athletes performed a running test at 80% of their *V*O_2max_ until exhaustion. The two tests were separated by 1 week (washout period), and on the following days, the athletes maintained the training routine. The tests were always performed at the same time in the morning and on the same treadmill (Movement LX 160 GII, São Paulo, Brazil) in a climatized environment with temperature and humidity controlled by a thermohygrometer (Incoterm, Porto Alegre, Brazil). To control the intensity of the exhaustion test, we used a cardiofrequencimeter (Polar FT1, Kempele, Finland) to monitor heart rate. The test was interrupted when the runner exhibited an inability to follow the treadmill’s speed in addition to verbal confirmation by the athlete and a reference between 19 and 20 on the Borg Rating of Perceived Exertion Scale [33]. The total run time was recorded.

### Biochemical analysis

The oxidant activity of malondialdehyde (MDA) was quantified in plasma by the reaction of thiobarbituric acid with the products of decomposition of hydroperoxides, according to the method described by Ohkawa et al. [34]. Total antioxidant capacity (TAC) was quantified in the plasma by measuring the scavenging activity of the free radical 2.2-diphenyl-1-picrylhydrazyl using the method described by Brand-Williams et al. [25]. Plasma concentrations of high-sensitivity C-reactive protein (hs-CRP) and alpha-1-acid glycoprotein (A1GPA) were quantified by immunoturbidimetry using specific commercial kits (Labtest) and an automatic analyzer (LabMax 240 Premium; Labtest) according to the manufacturer’s instructions. Creatine kinase (CK) was measured using the catalytic activity method, and concentrations of lactate dehydrogenase (LDH) were measured using the pyruvate-lactate method, both with specific commercial kits (Labtest, Minas Gerais, Brazil) in an automated analyzer (Labmax 240 Premium; Labtest, Minas Gerais, Brazil) according to the manufacturer’s instructions.

### Statistical analyses

Data are presented as the means ± standard deviations. Normality and homogeneity were evaluated using the Shapiro–Wilk and Levene tests, respectively. The results of the biomarkers were analyzed using a two-way ANOVA for multiple comparisons, with Tukey’s post hoc test, when appropriate. To compare the time-to-exhaustion running results, nutritional profile, mood, sleep quality, recovery/stress, perception of effort and biomarkers at baseline, we used a *t* test for paired samples. In addition, an individual analysis (simple subject analysis) was performed on the time variable of exhaustion to identify how many athletes improved their performance. Values of *p* < 0.05 were considered statistically significant. GraphPad Prism 7.0 (San Diego, Calif., USA) was used.

### Ethical statement

The study was fully conducted in accordance with the Declaration of Helsinki, its protocol was approved by the Research Ethics Committee of the Center for Health Sciences, Federal University of Paraiba (protocol n. 2.196.523). The participants signed an informed consent form according to Resolution 466/12 of the National Health Council (Brazil) and informed consent was obtained from all volunteers before the inclusion in the study.

## Results

### Characterization of purple grape juice and placebo drink

Table 1 presents the characteristics of the experimental beverages in terms of antioxidant activity and total phenolic composition. Purple grape juice had a higher presence of total phenolics and antioxidant activity than the placebo, which presented only small concentrations of antioxidants.

**Table 1:** Antioxidant activity of whole purple grape juice and the placebo drink

	Grape juice	Placebo
DPPH (µMol Trolox/mL)	13.0	8.1
ABTS (ATT µMol Trolox/mL)	9.5	ND
Total phenolics (mg/L)	3106.6	939.5
Flavanois (mg/L)	13.0	–
Flavonois (mg/L)	5.3	–
Phenolic Acids (mg/L)	83.8	1.6
Stilbenes (mg/L)	2.1	–

*DPPH* 2.2-diphenyl-1-picryl-hydrazyl, *ABTS* 2.2′-azinobis-3-ethylbenzthiazoline-6 sulfonic acid, *ND* not detected, *HPLC* high-performance liquid chromatography, *EMBRAPA* empresa brasileira de pesquisa agropecuária

### Characterization of participants

The participants presented cardiorespiratory fitness classified as excellent for active healthy men [35] but not for top-level athletes (Table 2). All of them practiced running at least four times a week, in addition to complementary activities such as resistance exercise (46.1%), soccer (15.4%) and cycling (23.1%), simultaneously with the running training. They participated in approximately 30 local or national running competitions per year.

**Table 2:** Baseline characteristics of runners (*n* = 14) (mean ± SD)

Age (years)	39 ± 9.2
BMI (kg/m^2^)	23.3 ± 2.3
Body fat (%)	10.5 ± 4.3
RHR (bpm)	53.7 ± 6.4
*V*O_2peak_ (mL/kg/min)	55.9 ± 6.5
Training (years)	10.7 ± 9.3
Training frequency (days/week)	5.0 ± 1.1
Training time (min/session)	65.0 ± 20.4
Training volume (km/week)	62.3 ± 18.9
Complementary activity (min/week)	148.9 ± 75.2
Work (h/days)	6.7 ± 2.5

*BMI* body mass index, *RHR* resting heart rate, *VO*_*2peak*_ peak oxygen consumption

Table 3 shows that athletes had the same physiological conditions on the two occasions in which they were tested after consuming the experimental juice or the placebo drink for biomarkers of oxidative stress, muscle soreness and inflammation. Psychometric tests indicated the same state of mood, stress, and recovery for training loads, as well as having slept for the same amount of time in the week prior to the two experimental procedures.

**Table 3:** Psychometric, sleep and recovery characteristics; biochemical markers; and nutritional status of the runners on the day of the experimental procedures (*n* = 14)

	Grape juice	Placebo	*p*
MDA (µmol/L)	4.3 ± 1.0	4.1 ± 1.0	0.99
TAC (%)	27.8 ± 7.6	27.8 ± 7.7	0.99
A1GPA (mg/dl)	70.4 ± 17.7	69.2 ± 14.2	0.70
hs-CRP (mg/dl)	1.5 ± 1.2	1.5 ± 1.6	0.95
CK (U/L)	193.2 ± 80.9	206.7 ± 118.0	0.99
LDH (U/L)	318.1 ± 65.0	309.5 ± 52.3	0.99
Energy (kcal/kg/day)	34.5 ± 12.6	38.9 ± 14.6	0.46
Carbohydrate (g/kg/day)	5.3 ± 1.8	5.5 ± 1.8	0.75
Protein (g/kg/day)	1.2 ± 0.4	1.3 ± 0.5	0.44
Fat (g/kg/day)	1.0 ± 0.5	1.3 ± 0.9	0.31
Vitamin A (RE/day)	509.5 ± 430.9	614.5 ± 372.7	0.54
Vitamin C (mg/day)	112.7 ± 130.3	129.9 ± 131.5	0.76
Vitamin D (mg/day)	28.6 ± 65.8	25.9 ± 80.9	0.93
Vitamin E (mg/day)	7.6 ± 5.3	12.7 ± 9.6	0.14
Copper (µcg/day)	0.9 ± 0.3	1.2 ± 1.2	0.39
Selenium (µcg/day)	45.9 ± 27.4	68.1 ± 21.6*	0.04
Zinc (mg/day)	6.2 ± 2.5	8.1 ± 4.3	0.21
Manganese (mg/day)	185.5 ± 76.8	183.5 ± 94.2	0.95
Sleep (h/day)	7.6 ± 1.7	7.4 ± 1.5	0.52
ESS-BR (score)	7.1 ± 2.2	5.8 ± 3.1	0.13
POMS (score)	89.9 ± 16.7	91.5 ± 12.4	0.56
Stress RESTQ-sport (score)	0.7 ± 0.6	0.7 ± 0.8	0.99
Recovery RESTQ-sport (score)	3.8 ± 0.8	3.9 ± 1.0	0.99

*MDA* malondialdehyde, *TAC* total antioxidant capacity, *A1GPA* alpha-1-acid glycoprotein, *hs*-*CRP* high-sensitivity c-reactive protein, *CK* creatine kinase, *LDH* lactate dehydrogenase, *ESS*-*BR* epworth sleepiness scale-brazilian, *POMS* profile of mood states; *RESTQ* sport recovery-stress questionnaire for athletes

*Indicates significant difference (*p* < 0.05) between the groups analyzed by paired *t* test

The nutritional conditions prior to the experiments were similar between the groups, except for the intake of the selenium mineral; a higher consumption was demonstrated prior to the intake of the placebo. Considering the reference values proposed by the International Society of Sports Nutrition—ISSN [36], the nutritional assessment revealed that runners consumed a low-calorie, normoglycidic, normolipidic and normoproteic diet at both timepoints. At both experiments, the participants presented low intakes of vitamins A and E, copper, zinc and manganese. Other micronutrients had adequate consumption levels. During the intervention period, the runners maintained their eating habits.

### Time-to-exhaustion running

**Fig. 3:**
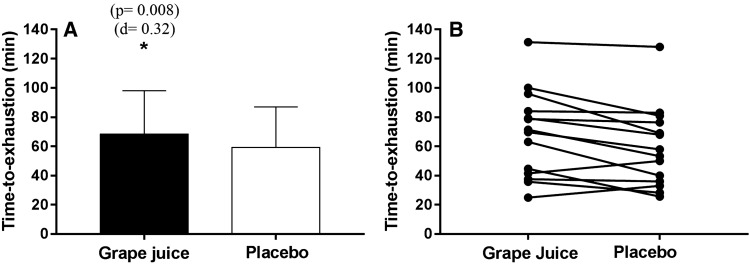
Effects of whole purple grape juice on the performance of the running test. **a** Physical performance (mean ± SD) and **b** individual results of each athlete’s absolute running time. *Indicates a significant difference (*p* < 0.05) between the groups analyzed by a paired *t* test (*n* = 14). *d *= small effect size

The results of the run to exhaustion test are shown in Fig. 3. In the running test until exhaustion (80% *V*O_2peak_) after placebo drink intake, athletes achieved a mean run time of 59.2 ± 27.8 min (Fig. 2a) and a mean distance traveled of 12.6 ± 6.3 km. In the procedure after purple grape juice ingestion, they presented a significantly higher performance that was 9.2 min longer (*p* = 0.008) in the mean running time-to-exhaustion, which represented an improvement of 18.7%, with a 1.9 km (*p* = 0.009) longer distance traveled when compared to that travelled by the placebo group. In Fig. 3b, a simple subject analysis is presented for the performance test of each athlete. It was observed that of the 14 runners evaluated, 12 of them presented better physical performance after consuming grape juice, and only two presented lower physical performance in the grape juice procedure.

### Oxidative stress

**Fig. 4:**
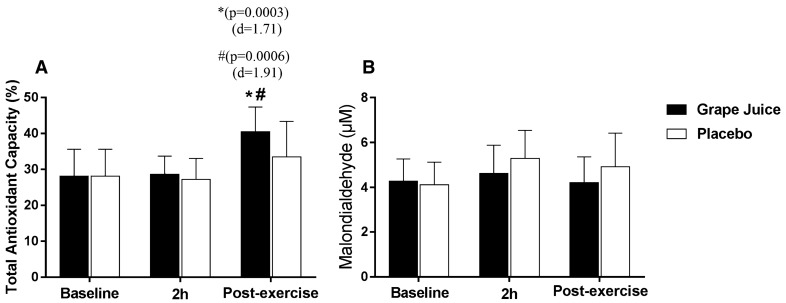
Effect of whole purple grape juice on oxidative stress biomarkers (mean ± SD). **a **Antioxidant activity and **b** lipid peroxidation in moments baseline, 2 h postsupplementation and post-exercise. Two-way ANOVA for repeated measurements (*n* = 14). *Indicates significant (*p* < 0.05) intragroup difference between the baseline and postexercise timepoints. ^#^Indicates significant (*p* < 0.05) intragroup difference between the 2 h and postexercise timepoints. *d *= large effect size

The oxidative stress parameters analyzed are presented in Fig. 4. Analysis of the time-group interaction indicated that greater antioxidant activity occurred with the time factor at the postexercise timepoint (43.6% increase; *p* = 0.0003) when compared to the 2 h postsupplementation timepoint (increase of 1.7%; *p* > 0.05) in relation to the baseline values in the procedure with the purple grape juice (Fig. 4a). Despite this pronounced increase, no difference was found in the interaction group. On the other hand, there were no changes in the lipid peroxidation response in any of the procedures (Fig. 4b).

### Inflammation and muscle damage

**Fig. 5:**
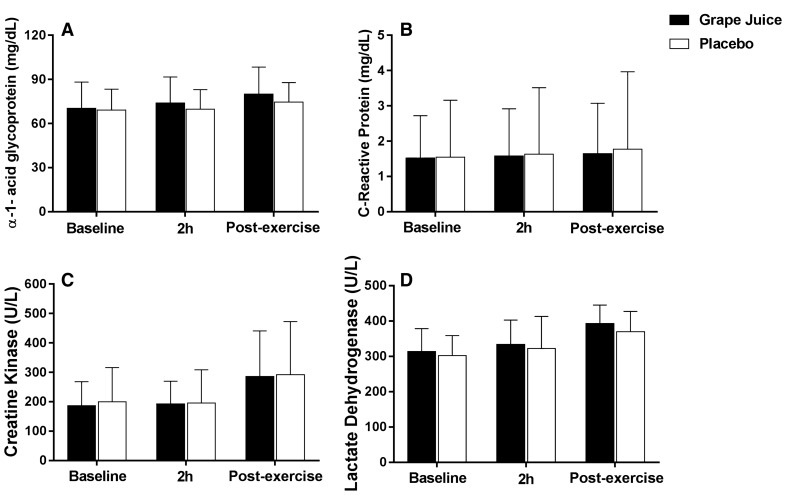
Effect of whole purple grape juice on inflammatory and muscle wasting biomarkers (mean ± SD). **a** A1GPA and **b** hs-CRP; **c** CK and **d** LDH in moments baseline, 2 h postsupplementation and post-exercise. Two-way ANOVA for repeated measurements (*n* = 14)

In Fig. 5, the data demonstrate the acute inflammatory response assessed by the A1GPA and PCR markers (panels A and B), and the muscle damage activity assessed by the CK and LDH enzymes were not significantly modified (*p* = 0.99) at any timepoint (panels C and D).

## Discussion

The present study demonstrated that a single dose of purple grape juice (10 mL/kg of body weight) promoted increased plasma antioxidant activity and significant improvements in specific physical performance in recreational runners.

Previous studies in animal models have demonstrated the ergogenic effects of purple grapes and their derivatives including antioxidant protection [6, 9, 11, 37] and improvements in physical performance [8, 9, 38]. However, studies with humans on this topic are still scarce. In addition, the studies that demonstrated an ergogenic effect of supplementation administered grape polyphenol concentrate [16, 39], and only one study considered food intake [13]. Lafay et al. [39] supplemented athletes with grape extract (400 mg/day) for 30 days and observed improvement in physical performance and antioxidant capacity and reduction in muscle soreness. Improvement in physical performance was also observed by Deley et al. [16] when they tested the intake of a single dose of 500 g of polyphenol in active adults and observed greater time to exhaustion, time to reach maximal perceived exertion and half-recovery time for *V*O_2max_.

Until the present study, the ergogenic capacity of grape juice in humans had been demonstrated only by Toscano et al. [13], who chronically supplemented athletes and observed increased physical performance in running associated with improved antioxidant activity. This is very relevant data for athletes, but the present study shed light on even more important data by demonstrating that only a single dose of whole grape juice is able to promote better results than those observed with multiple-dose supplementation. The magnitude of the increase in the physical performance of the runners found by Toscano et al. [13] after 28 days of supplementation was approximately 15%, whereas that observed in this study was 18.7% for a single dose. These data demonstrate an important practical implication, that athletes do not necessarily need to drink multiple doses of grape juice, but only a single dose at pre-exercise time.

This single-dose response is supported by other studies with grape derivatives and other foods. Acute supplementation with grape and apple polyphenols [16] improved in time to exhaustion by 9.7% and pomegranate extract [40] also improved time to exhaustion in 12%, both in physically active individuals. *Ecklonia cava* polyphenol [15] besides improved exhaustion time in college students by a greater magnitude (30%) than that observed in the present study, increased the *V*O_2max_ in 6.5%. Studies suggest that these variations in supplementation responses may be explained by the time the supplement was consumed. The time between ingestion and exercise in these studies varied from 30 min to 1 h pre-exercise. According to Stalmach et al. [41] and Keane et al. [42], antioxidant responses from phenolic metabolites peak in plasma concentrations between 1 and 2 h after food intake.

Nutritional strategies to promote the athlete’s maximum performance and a better recovery and to increase their usual energy intake, especially in the form of carbohydrates, are important during training sessions or precompetition [36, 43]. From a new perspective, the present study indicates that endurance athletes can also benefit from the intake of preworkout grape juice. It is plausible to suppose that the increase in physical performance could have been promoted by the rich carbohydrate composition of the juice (32 g of carbohydrates/200 mL of grape juice), which would corroborate the previous data that demonstrated the benefit of a high-carbohydrate diet precompetition [44, 45]. However, the placebo drink offered in the control session, along with the pretest meal, had a very similar amount of carbohydrates, providing approximately 1.9 g/kg of carbohydrate (considering a 70 kg athlete) 2 h before the test according to the American College of Sports Medicine [43] guidelines for sports nutrition. Thus, we can state from our findings that the addition of precompetition whole purple grape juice promoted additional nutritional benefit to recreational-level runners.

The mechanism of muscle fatigue for long-term exercise is not fully understood in the literature, but it is known that metabolic acidosis and glycogen depletion are well established causes [46]. Once the placebo drink in this study was isoglycidic, we cannot attribute the ergogenic effect of grape juice to carbohydrates. On the other hand, Jing-Jing et al. [47] indicate that oxidative stress hinders the mechanism of muscle contraction, so we can assume that antioxidant compounds may retard this mechanism of fatigue. However, further studies are needed to confirm this mechanism or even to determine other components involved in delaying polyphenol fatigue. Additionally, the polyphenols present in grapes also have anti-inflammatory [13, 48] and vasodilator properties [49, 50], which may contribute to increase the supply of muscle oxygen.

Athletes presented inadequate dietary intake of antioxidant nutrients, such as vitamins A and E, in addition to the minerals zinc, copper and manganese [36]. This condition leads the athlete to a state of oxidative imbalance and may decrease the protective activity of antioxidant enzymes (superoxide dismutase, glutathione and catalase), which is caused mainly by the low intake of minerals (copper, zinc and manganese), which are considered enzyme binding cofactors responsible for the first-line defense in the antioxidant system, superoxide dismutase [51–53]. In this sense, due to the rich composition of vitamins [54], minerals [55] and polyphenolic compounds [56, 57], the grape juice may have momentarily adjusted the athletes’ nutritional deficiency. To better assess this possibility, it is necessary to study athletes with an adequate nutritional intake.

In the present study, we can declare that a limited antioxidant effect was observed since we evaluated only TAC to assess antioxidant activity, and this parameter increased after the exhaustion test, without changing the plasma concentrations of lipid peroxidation by MDA. However, this effect may be explained by the bioavailability of polyphenols, confirmed by plasma concentrations after 2–3 h of juice ingestion [58, 59]. In fact, we observed the highest antioxidant concentration after approximately 3 h since the exhaustion test occurred after 2 h of juice intake and had an average duration of 68 min. The lack of reduction in lipid peroxidation observed in this study was also demonstrated in other studies that performed a similar exercise protocol at a similar intensity and also used antioxidant supplementation [60, 61].

A practical implication of this study is the recommendation of whole grape juice as a potentially ergogenic food for recreational athletes. Grape juice may be considered an interesting option for athletes to consume daily or even ingest in the hours before a competition. Grape juice may be considered a complete preworkout food for athletes because it presents high caloric value from its composition of carbohydrates and still contains several antioxidants capable of delaying fatigue and optimizing recovery.

The study presented important limitations regarding the plasma antioxidant response since it evaluated only TAC but not antioxidant enzymes. In addition, although MDA is a well-accepted biomarker in the literature, it evaluates nonspecific cell oxidation. Given these limitations, we suggest that studies with a similar protocol be developed to evaluate antioxidant enzymes and specific oxidative stress markers. Thus, it will be possible to demonstrate the protective effect of purple grape juice in an even more important way.

We also suggest developing new studies that include high-performance athletes since this study allows us to consider the results only for recreational athletes. Since in this study, 2 h after the ingestion of grape juice, there was no increase in plasma concentrations of antioxidants, we suggest a new study that evaluates the physical performance after 3 h of juice intake, following our same protocol.
