# Correction to: A single dose of purple grape juice improves physical performance and antioxidant activity in runners: a randomized, crossover, double-blind, placebo study

**DOI:** 10.1007/s00394-020-02453-4

**Published:** 2020-12-23

**Authors:** Lydiane de Lima Tavares Toscano, Alexandre Sérgio Silva, Ana Carla Lima de França, Bruno Rafael Virgínio de Sousa, Eder Jackson Bezerra de Almeida Filho, Matheus da Silveira Costa, Aline Telles Biasoto Marques, Darcilene Fiuza da Silva, Klécia de Farias Sena, Gilberto Santos Cerqueira, Maria da Conceição Rodrigues Gonçalves

**Affiliations:** 1 Programa de Pós-graduação em Ciências da Nutrição, Universidade Federal da Paraíba (UFPB), João Pessoa, Paraíba Brazil; 2 Laboratório de Estudos do Treinamento Físico Aplicado ao Desempenho e a Saúde, Departamento de Educação Física, Universidade Federal da Paraíba (UFPB), Centro de Ciências da Saúde, Campus I, Cidade Universitária, João Pessoa, Paraíba CEP 58059-900 Brazil; 3 Empresa Brasileira de Pesquisa Agropecuária, Embrapa Semiárido, Petrolina, Pernambuco Brazil; 4 Faculdade de Farmácia, Universidade Federal da Bahia, Salvador, Bahia Brazil; 5 Departamento de Morfologia, Universidade Federal do Ceará, Fortaleza, Ceará Brazil

## Correction to: European Journal of Nutrition (2020) 59:2997–3007 10.1007/s00394-019-02139-6

The article A single dose of purple grape juice improves physical performance and antioxidant activity in runners: a randomized, crossover, double‑blind, placebo study, written by Lydiane de Lima Tavares Toscano, Alexandre Sérgio Silva, Ana Carla Lima de França, Bruno Rafael Virgínio de Sousa, Eder Jackson Bezerra de Almeida Filho, Matheus da Silveira Costa, Aline Telles Biasoto Marques, Darcilene Fiuza da Silva, Klécia de Farias Sena, Gilberto Santos Cerqueira and Maria da Conceição Rodrigues Gonçalves, was originally published Online First without Open Access. After publication in volume 59, issue 7, page 2997–3007 the author decided to opt for Open Choice and to make the article an Open Access publication. Therefore, the copyright of the article has been changed to © The Authors 2020 and this article is licensed under a Creative Commons Attribution 4.0 International License, which permits use, sharing, adaptation, distribution and reproduction in any medium or format, as long as you give appropriate credit to the original author(s) and the source, provide a link to the Creative Commons licence, and indicate if changes were made. The images or other third party material in this article are included in the article’s Creative Commons licence, unless indicated otherwise in a credit line to the material. If material is not included in the article’s Creative Commons licence and your intended use is not permitted by statutory regulation or exceeds the permitted use, you will need to obtain permission directly from the copyright holder. To view a copy of this licence, visit https://creativecommons.org/licenses/by/4.0/.

The original article has been corrected.

